# Utility of BeBack crossing catheter in fistula creation during percutaneous deep vein arterialization

**DOI:** 10.1016/j.jvscit.2023.101201

**Published:** 2023-04-29

**Authors:** Daniel Yuxuan Ong, Enming Yong, Uei Pua

**Affiliations:** a Department of Diagnostic Radiology, Tan Tock Seng Hospital, Singapore; b Department of General Surgery, Tan Tock Seng Hospital, Singapore

**Keywords:** BeBack crossing catheter, Chronic limb-threatening ischemia, CLTI, Endovascular, PAD, pDVA, Percutaneous deep vein arterialization, Peripheral arterial disease

## Abstract

For patients with “no-option” chronic limb-threatening ischemia, a final attempt can be made for limb salvage in the form of percutaneous deep vein arterialization (pDVA). In the present study, we describe five cases of pDVA using a BeBack crossing catheter (Bentley InnoMed GmbH; previously, the GoBack crossing catheter; Upstream Peripheral Technologies). From November 2021 to July 2022, five patients underwent pDVA using the BeBack crossing catheter. The demographic data, procedural details, and patient outcomes were recorded. Successful vascular crossing was achieved in all five cases using the BeBack device. No surgical complications were encountered. The limb salvage rate was 60%, and the wound closure rate was 40%. No mortalities occurred during the follow-up period. The findings from the present study demonstrate that the use of the BeBack crossing catheter for pDVA is safe and feasible.

Deep venous arterialization (DVA) represents a final attempt at limb salvage before major amputation becomes necessary.^1^ During the past decade, percutaneous DVA (pDVA) using a propriety device (LimFLow; LimFlow Inc) has been shown to be feasible, with respectable outcomes for the “no-option” chronic limb-threatening ischemia (CLTI) population.^2^^,^^3^

In the present study, we describe five cases of pDVA using the BeBack crossing catheter (Bentley InnoMed GmbH; previously, the GoBack crossing catheter; Upstream Peripheral Technologies; Fig 1). The BeBack crossing catheter is a single-lumen crossing catheter with a directionally controlled, curved nitinol needle tip. The needle has an adjustable protrusion length from a straight (2-3 mm) to a fully curved (11 mm) position. The directionally controlled needle can be deployed to various lengths and angulation. It supports 0.018-in. and 0.014-in. catheter guidewires for the 4F and 2.9F versions, respectively. The device is useful for both intraluminal crossing and subintimal reentry.

**Fig 1:**
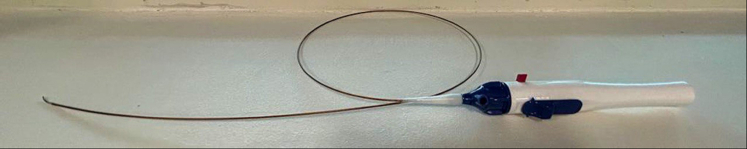
BeBack crossing catheter (Bentley InnoMed GmbH; previously, the GoBack crossing catheter; Upstream Peripheral Technologies).

## Case report

This report fulfills our institution's criteria for an ethics board waiver. Using our institution's electronic medical records, five patients with “no-hope” CLTI who underwent pDVA from November 2021 to July 2022 using the BeBack crossing catheter were identified. All five patients provided written informed consent for the report of their case details and imaging studies.

### Operative technique

For intraoperative anticoagulation, the patients received an initial dose of heparin at 75 U/kg, with a 1000-U bolus every hour. Under ultrasound guidance, the common femoral artery was accessed. Simultaneous arteriography and venography were performed with contrast administration via injection through an antegrade femoral arterial sheath (long 6F or 7F sheath) and a retrograde venous sheath (5F radial sheath) placed in the distal posterior tibial vein (PTV) to delineate the segment of the posterior tibial artery and PTV in proximity for easy arteriovenous (AV) crossing.

**Fig 2:**
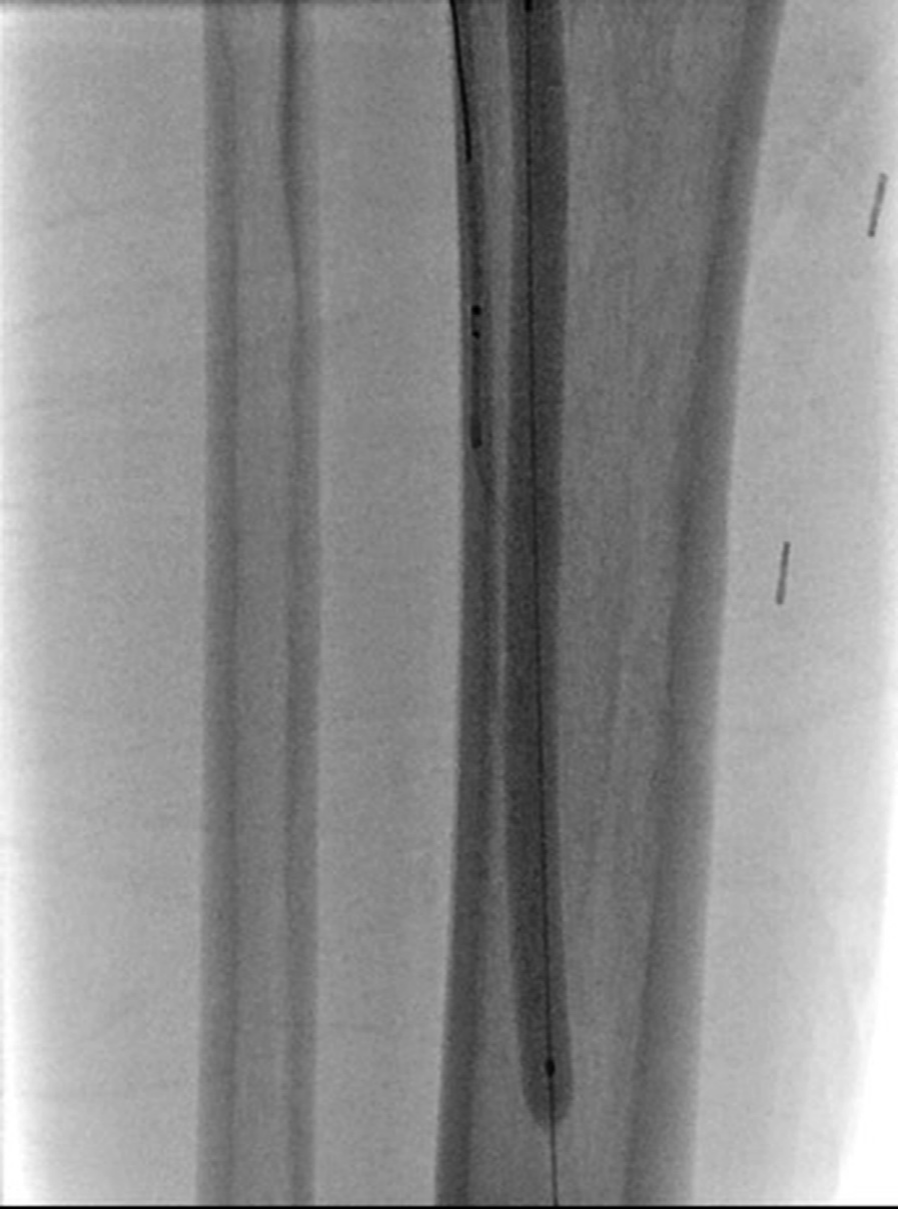
Intraoperative fluoroscopic image demonstrating the ruptured venous balloon target, with crossing of the wire from the posterior tibial artery into the posterior tibial vein (PTV).

The BeBack crossing catheter was used as the reentry device to target an inflated balloon (5 mm × 100 mm) within the PTV introduced via the venous sheath. The “C-marker” of the BeBack device was aligned tangential to the balloon, followed by needle puncture of the mid-balloon (Fig 2; Supplementary Video, online only). An inflated venous balloon has the advantage of providing a large three-dimensional target, allowing for direct visualization of balloon rupture to confirm venous entry, and the ability to shuttle the crossing wire down to the level of the venous sheath within the punctured balloon chamber.

Once the AV fistula (AVF) was created, antegrade wiring down the PTV to reach the lateral plantar vein was performed. Crossing the venous valves can be achieved using a diagnostic 4F Berenstein catheter (Tempo Aqua; Cordis Corp), combined with a 0.014-in. or 0.018-in. Nitinol guidewire with high tip retention property (eg, Command wire; Abbott Vascular), using the “dancing wire technique.”^3^

**Fig 3:**
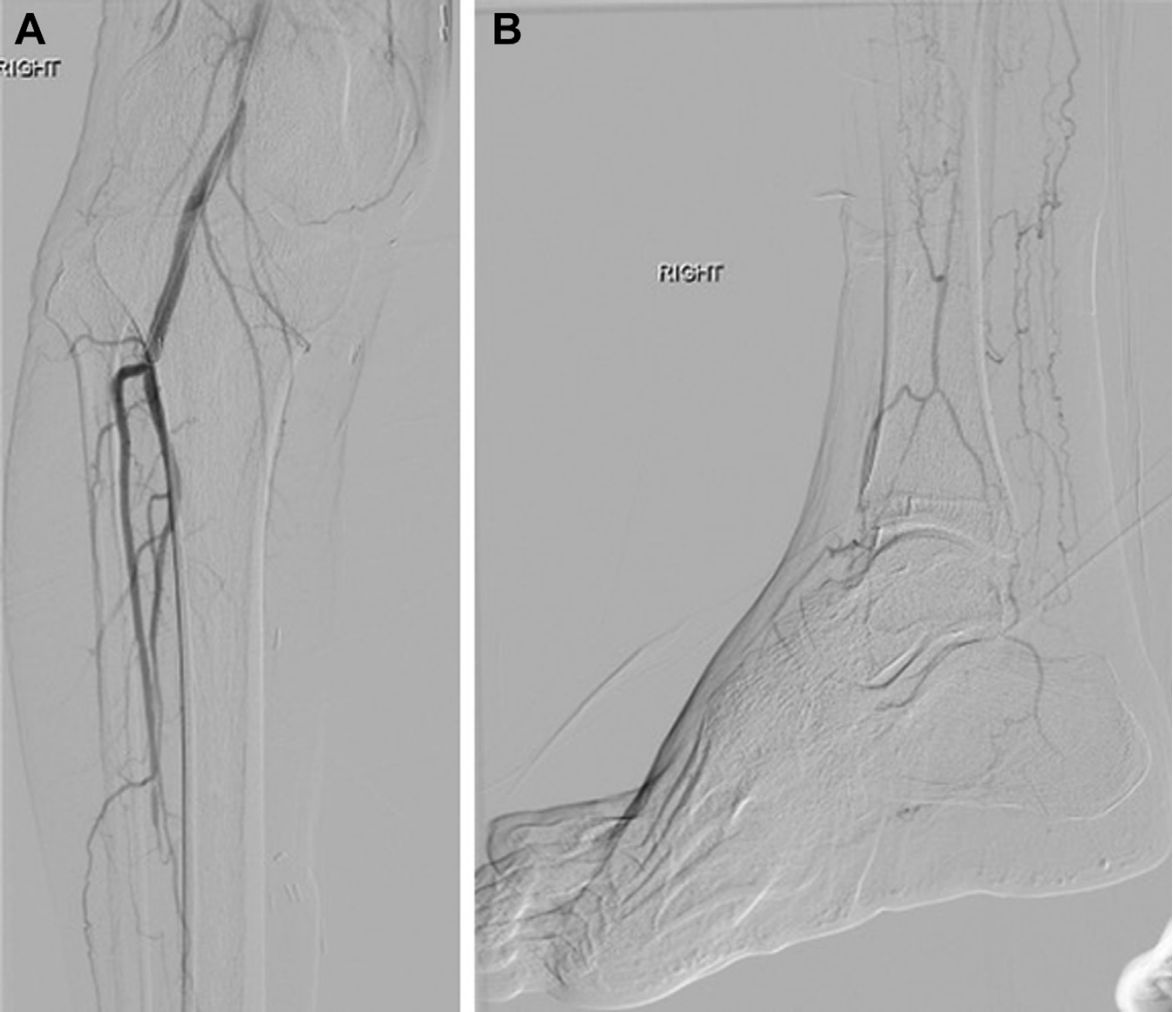
**A** and **B,** Intraoperative angiographic images demonstrating diffuse atherosclerotic disease in the lower limb arteries.

**Fig 4:**
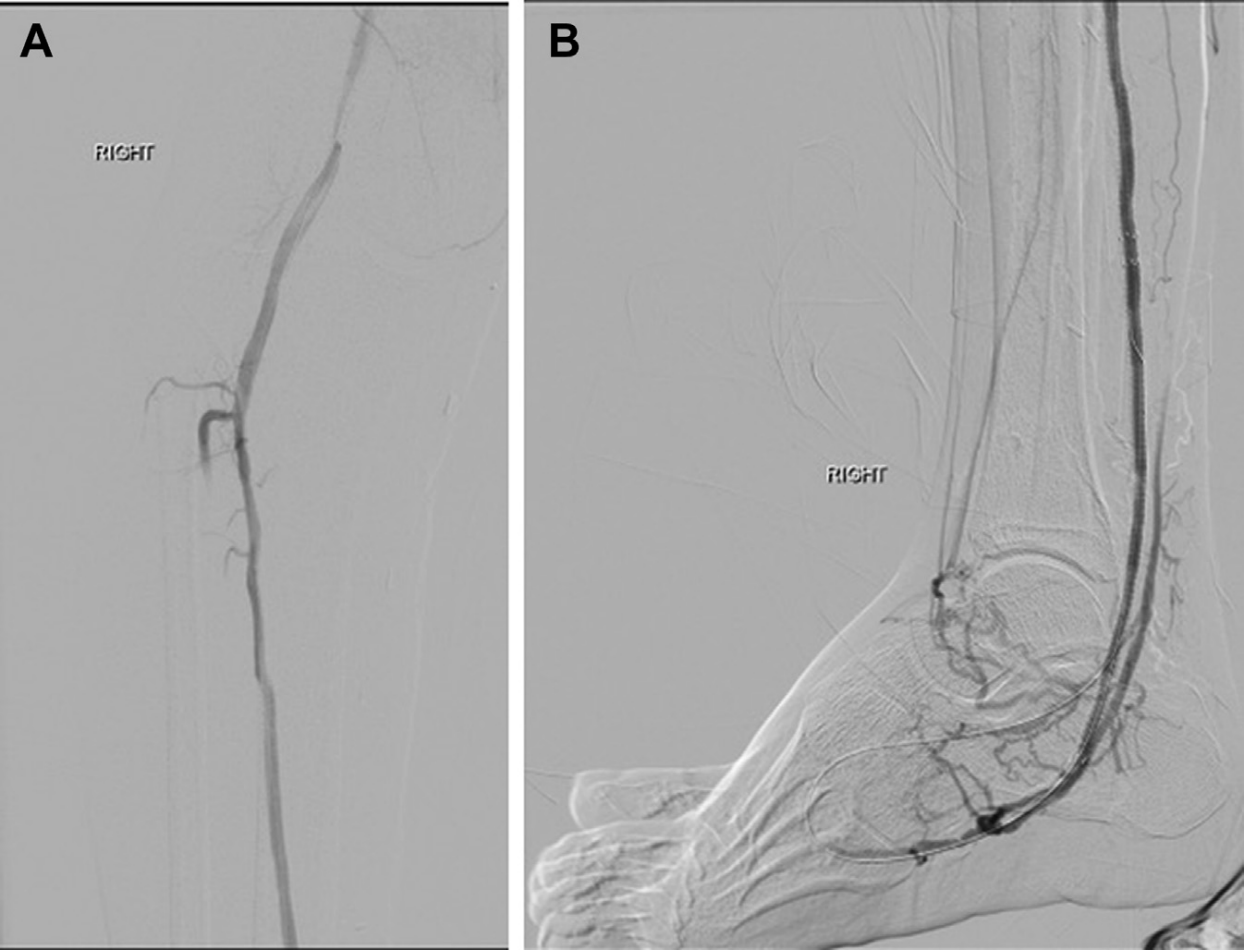
**A** and **B,** Intraoperative angiographic images demonstrating technically successful percutaneous deep vein arterialization (pDVA).

In the infrapopliteal venous segment, deployment of stent grafts (eg, Viabahn; W.L. Gore & Associates) renders the venous valves incompetent and also covers the venous perforators, which could result in steal syndrome. Retrograde stent grafting typically extends from the mid-calcaneum in an overlapping fashion across the AVF into the supplying artery and postdilated with 4- to 5-mm angioplasty balloons. For the inframalleolar venous segment, balloon valvulotomy was performed using 3- to 4-mm angioplasty balloons or semi-inflated cutting balloons. Further focalization of flow (ie, opacification of the forefoot veins) can be performed by embolization of the perforator veins ∼3 to 4 weeks after the procedure. Angiographic images were recorded before and after the procedure to document technical success (Figs 3 and 4).

## Results

The demographic information of the five patients (four men and one woman; mean age, 64 years; range, 58-70 years) is included in Table I. The procedural details are presented in Table II, and the preoperative clinical parameters are listed in Table III.

**Table I:** Patient demographics

Pt. No.	Age, years	Gender	Medical history
1	68	Male	Peripheral vascular disease, hypertension, alcohol abuse
2	70	Male	Peripheral vascular disease, diabetes, hyperlipidemia, hypertension, alcohol abuse
3	58	Male	Peripheral vascular disease, diabetes, hyperlipidemia, hypertension
4	60	Male	Peripheral vascular disease, diabetes, hyperlipidemia, hypertension, ischemic heart disease
5	64	Female	Peripheral vascular disease, diabetes, hypertension, end-stage renal failure, anemia

*Pt. No.,* Patient number.

**Table II:** Procedural details

Pt. No.	Venous puncture site	Arterial puncture site	BeBack crossing catheter outcome	Technical success of DVA	Surgical complications	Major amputation	Wound closure	Perioperative mortality
1	PTV; plantar sole approach	Common femoral artery	Success	Partial success: side branches demonstrated on completion angiography; repeat angiography performed 1 month later, for possible embolization of side branches, which revealed achievement of full arterialization, with no intervention required	No	Below knee amputation 8 months after surgery	NA	No
2	Right medial plantar vein	Common femoral artery	Success	Success	No	No	Closure at 12.7 months	No
3	Distal PTV	Common femoral artery	Success	Success	No	Below knee amputation 1 month after surgery due to overwhelming foot sepsis	NA	No
4	Lateral plantar vein	Common femoral artery	Success	Partial success: focal recalcitrant stenosis encountered in distal PTV; attempts at balloon angioplasty failed, with multiple balloon ruptures; 1 week later, the stenosis was successfully ameliorated using an ultra-high-pressure balloon and external picking technique	No	No	Reduction in wound size of 75%, with 1.5 × 1-cm wound remaining	No
5	Plantar vein access	Common femoral artery	Success	Partial success: completion angiography demonstrated partial arterialization; repeat angiography 3 weeks later demonstrated complete arterialization; at the same session, embolization of a medial marginal vein and large lateral tarsal vein was performed for focalization of DVA in the foot	No	No	Closure at 4.7 months	No

*DVA,* Deep vein arterialization; *NA,* not available; *Pt. No.,* patient number; *PTV,* posterior tibial vein.

**Table III:** Clinical parameters

Pt. No.	Preoperative Rutherford classification	Preoperative WIfI stage
1	5	W, 1; I, 2; fI, 3; overall, 4
2	5	W, 2; I, 3; fI, 2; overall, 4
3	6	W, 3; I, 1; fI, 3; overall, 4
4	5	W, 2; I, 2; fI, 0; overall, 3
5	5	W, 2; I, 1; fI, 0; overall, 3

*fI,* Foot infection; *I,* ischemia; *Pt. No.,* patient number; *W,* wound; *WIfI,* wound, ischemia, foot infection.

All patients had successful crossing with the BeBack device with formation of DVA. No postoperative complications were encountered in any of the cases. Three of the five patients had partial technical success. Two cases of limb loss occurred. Patient 1 underwent a below knee amputation at ∼8 months after DVA creation, after occlusion of the DVA stent. Patient 3 underwent a below knee amputation at ∼1 month after DVA creation because of severe rest pain from forefoot and hindfoot gangrene, despite a vascular duplex ultrasound scan demonstrating patent DVA. Patient 4 developed DVA occlusion ∼8 months after the procedure. Overall, the limb salvage rate was 60%, and the wound closure rate was 40%. No mortalities occurred during the follow-up period.

## Discussion

Patients with CLTI experience increased rates of lower limb amputation. DVA provides a viable treatment option for “no-option” CLTI, with limb salvage rates of ≤75% at 12 months.^1^ Percutaneous femoropopliteal bypass using the PQ Bypass detour system (PQ Bypass) is a promising treatment alternative for patients with long-segment femoropopliteal lesions. However, percutaneous femoropopliteal bypass was beyond the scope of our technical report.

A Singapore pilot study using a proprietary endovascular pDVA device reported a 100% technical success rate, with no above ankle amputations, deaths, or major reinterventions at 30 days.^3^ Although a dedicated device for pDVA will allow for a more standardized procedure, access to the proprietary device remains limited outside the setting of clinical trials. Off-the-shelf pDVA, therefore, represents a viable option.

The Japan DEPARTURE (deep venous Arterialization procedure for patients with no-option chronic limb-threatening ischemia in Japan) study demonstrated safe and efficacious results of pDVA for patients with no-option CLTI.^4^ Our study differs in that they used various techniques, including a venous arterialization simplified technique, a modified venous arterialization simplified technique, an AV-spear technique, and a reentry device for AVF creation.

Compared with other devices, such as the Outback Re-Entry Catheter (Cordis), we found that the long, directionally controlled, curved needle tip of the BeBack crossing catheter is a unique feature that allows for reentry with comparative ease. In the present study, we demonstrated that the use of the BeBack crossing catheter in pDVA is safe and feasible, with successful vascular crossing in all five cases, with no surgical complications encountered. The various technical success rates reflect the infancy of pDVA and the associated learning curve.

## Conclusions

In the present study, we have demonstrated that the use of the BeBack crossing catheter for pDVA is safe and feasible.

## Appendix (online only)

Supplementary Video (online only) Intraoperative fluoroscopic video clip demonstrating rupture of the venous balloon target via puncture by the BeBack needle, with subsequent crossing of the wire from the posterior tibial artery into the posterior tibial vein (PTV).
